# Dexamethasone Pretreatment Alleviates Isoniazid/Lipopolysaccharide Hepatotoxicity: Inhibition of Inflammatory and Oxidative Stress

**DOI:** 10.3389/fphar.2017.00133

**Published:** 2017-03-15

**Authors:** Hozeifa M. Hassan, Hongli Guo, Bashir A. Yousef, Ding Ping-Ping, Luyong Zhang, Zhenzhou Jiang

**Affiliations:** ^1^Jiangsu Key Laboratory of Drug Screening, China Pharmaceutical UniversityNanjing, China; ^2^Department of Pharmacology, Faculty of Pharmacy, University of GeziraWad-Medani, Sudan; ^3^Department of Pharmacology, Faculty of Pharmacy, University of KhartoumKhartoum, Sudan; ^4^Jiangsu Center for Pharmacodynamics Research and Evaluation, China Pharmaceutical UniversityNanjing, China; ^5^Key Laboratory of Drug Quality Control and Pharmacovigilance (China Pharmaceutical University), Ministry of EducationNanjing, China

**Keywords:** isoniazid, lipopolysaccharide, hepatotoxicity, dexamethasone, CYP2E1, inflammatory stress, oxidative stress

## Abstract

Isoniazid (INH) remains a cornerstone key constitute of the current tuberculosis management strategy, but its hepatotoxic potentiality remains a significant clinical problem. Our previous findings succeed to establish a rat model of INH hepatotoxicity employing the inflammatory stress theory in which non-injurious doses of inflammatory-mediating agent bacterial lipopolysaccharides (LPS) augmented the toxicity of INH that assist to uncover the mechanisms behind INH hepatotoxicity. Following LPS exposure, several inflammatory cells are activated and it is likely that the consequences of this activation rather than direct hepatocellular effects of LPS underlie the ability of LPS to augment toxic responses. In this study, we investigated the potential protective role of the anti-inflammatory agent dexamethasone (DEX), a potent synthetic glucocorticoid, in INH/LPS hepatotoxic rat model. DEX pre-treatment successfully eliminates the components of the inflammatory stress as shown through analysis of blood biochemistry and liver histopathology. DEX potentiated hepatic anti-oxidant mechanisms while serum and hepatic lipid profiles were reduced. However, DEX administration was not able to revoke the principal effects of cytochrome P450 2E1 (CYP2E1) in INH/LPS-induced liver damage. In conclusion, this study illustrated the DEX-preventive capabilities on INH/LPS-induced hepatotoxicity model through DEX-induced potent anti-inflammatory activity whereas the partial toxicity seen in the model could be attributed to the expression of hepatic CYP2E1. These findings potentiate the clinical applications of DEX co-administration with INH therapy in order to reduce the potential incidences of hepatotoxicity.

## Introduction

Although isoniazid (INH) is a key component of the current tuberculosis management strategy, but its hepatotoxic potentiality remains a significant clinical problem (Yew and Leung, 2006; Metushi et al., 2011). Despite extensive research efforts for understanding the reasons behind INH-induced liver toxicity, the exact underlying mechanisms are poorly comprehended (Wang et al., 2016). As a part of the persistent efforts to uncover the INH hepatotoxic mechanisms, our previous work succeeds to establish a 2-week INH-induced hepatotoxicity model (Hassan et al., 2016). In that model, bacterial lipopolysaccharide (LPS), a Gram-negative bacterial outer cell wall’s membrane constituent, clearly augmented the INH-induced liver injury. The major theory behind this enhancement activity was inflammatory stress theory, in which an inflammatory-mediating agent caused an incidence of systemic inflammation that might reduce xenobiotic toxicity threshold therefore, intensify drug-induced toxicity (Yee et al., 2000; Buchweitz et al., 2002; Deng et al., 2009). Moreover, most of patients clinically suffering from INH-induced hepatotoxicity show liver biopsy findings including necrosis and inflammation which is mostly associated with eosinophils infiltration (Bjornsson et al., 2007). Episodes of modest inflammation, although benign on their own, are probably commonplace in people and have the ability to augment the toxicities of several xenobiotic agents (Liu et al., 2000; Lu et al., 2011). Indeed, it has been suggested that exposure to endogenous LPS due to increased movement of bacteria across a compromised intestinal mucosa contributes to the hepatotoxicity produced by various agent (Brown et al., 1996; Barton et al., 2000; Yee et al., 2000). Moreover, LPS influences the pharmacokinetics of drugs (Ma et al., 2011) by modulating the activities of both phase I (Pan et al., 2003; Xu et al., 2006) and phase II (Richardson et al., 2006; Alkharfy et al., 2008) drug-metabolizing enzymes. LPS influence INH-induced hepatotoxicity by affecting INH pharmacokinetics through LPS ability to suppress the protein translation and mRNA transcription of cytochrome P450 isozymes, including cytochrome P450 2E1 (CYP2E1; Hasegawa et al., 1999). Co-administration of INH and LPS caused serious liver injury and mixed hepatotoxicity in rats, including hepatocellular injury and cholestasis (Su et al., 2014), which augments the role of inflammation in INH-induced hepatotoxicity. These results raise the possibility that a mild episode of inflammation might render human patients susceptible to INH-induced liver injury.

Furthermore, the key role played by the over-production of both CYP2E1 and reactive oxygen species (ROS) have also been noticed, in which liver susceptibility toward inflammatory mediators toxicity is elevated due to CYP2E1 over-expression. In addition to increased liver ROS production, both LPS and INH amplifies CYP2E1 production, which in returns exaggerates their tissue damage capabilities (Lu and Cederbaum, 2010; Aubert et al., 2011; Cederbaum et al., 2012).

For more than half a century, corticosteroids including dexamethasone (DEX), a synthetic glucocorticoid, have been used as a potent anti-inflammatory and immunosuppressive agents in treatment of different inflammatory disorders (Pang et al., 2012; Du et al., 2014). DEX also has anti-endotoxin activity through intervening with pro-inflammatory mediators’ synthesis mechanisms (Yubero et al., 2012; Wang et al., 2013). Therefore, our main goals in the present study are to alleviate the augmenting effects of LPS in INH-mediated liver injury through attenuation of the inflammatory stress by DEX administration and to find out the possible mechanisms behind DEX protection when co-administered with INH/LPS.

## Materials and Methods

### Drugs and Reagents

INH (Lot number MKBV9475V, analytical standard ≥99%), LPS (Lot number 025M4128V, derived from *Escherichia coli* 0128:B12 serotype, source strain is CDC 2440-69), were purchased from Sigma-Aldrich (St. Louis, MO, USA). DEX as sodium phosphate ready-made injections (Lot number 5160303) was obtained from Hubei Chang Tian Pharmaceutical Company (Hubei, China). TRIzol reagent bought from Invitrogen Life Technologies (CA, USA) while PrimeScript^TM^ RT Master Mix from Takara Biotechnology (Dalian, China) and SYBR Green Supermix from Vazyme Biotech (Nanjing, China). Other reagents were from high-analytical grade commercially available or as specified in the relevant contexts.

### Animals

Male, Sprague-Dawley rats weighing 200–220 g were obtained from Shanghai Lingchang Biological Technology Co., Ltd. (Shanghai, China). All experimental procedures were conducted in accordance with the guide for Institutional Animal Care and Use Committee at China Pharmaceutical University and the National Institutes of Health (NIH) guidelines for the care and use of laboratory animals. Rats were housed in controlled environmental conditions (23 ± 1°C, 55 ± 5% relative humidity, 12 h light–dark cycle) with free access to food and water *ad libitum*. Animals were acclimatized for 1 week before conducting the experiments. All experimental procedures were approved by China Pharmaceutical University, National Drug Screening Centre and Jiangsu Institute of Materia Medica ethical committees, Nanjing, China.

### Experimental Design and Treatment Schedule

Rats (*n* = 8 per group) were randomly divided into five groups: control group (group I), INH 200 mg/kg (ig plus intravenous LPS 2 mg/kg (group II), INH 400 mg/kg (ig) plus intravenous LPS 2 mg/kg (group III), while both group IV and group V received the same INH and LPS doses in addition to intraperitoneal DEX (4 mg/kg).

INH was administered for 14 consecutive days, whereas LPS was given as bolus dose at day 14, 2 h before the last INH dose. DEX was also given as a single dose at day 14, 1 h prior LPS administration. These selected concentrations were in accordance with previous research on INH/LPS combinations (Clayton et al., 2007; Metushi et al., 2012; Su et al., 2014; Hassan et al., 2016) and DEX (Dandona et al., 1999; Eum et al., 2003; Cui et al., 2015). Rats were sacrificed after INH last dose; blood was collected, allowed to clot at room temperature and centrifuged for serum. Liver sections were isolated, frozen in liquid nitrogen then stored for further experiments.

### Serum and Liver Biochemistry

For hepatotoxicity determination, following the standard enzymatic techniques, serum alanine transaminase (ALT), aspartate transaminase (AST), total bile acids (TBA), total bilirubin (TBil), gamma-glutamyl transferase (GGT), triglyceride (TG), and total cholesterol (TC) levels were measured by HITAC7170A automatic analyzer (Hitachi, Japan). Both liver TG and TC were determined following the manufacturer’s instructions of corresponding detection kits (Nanjing Jiancheng Bioengineering Institute, Nanjing, China).

### Histopathological Analysis

Immediately following sacrifice of the rats, histological examinations were conducted on rat liver slices. Liver slices were directly fixed in 10% paraformaldehyde solution and embedded in paraffin wax, then stained with hematoxylin and eosin (H&E). Slides were coded, randomized, and assessed by pathologists who during the evaluation of the slides were blinded to the treatment groups.

### Oil Red O Staining

In order to verify accumulation of lipids in hepatic tissues following INH/LPS co-treatment, fresh frozen liver sections were treated with oil red O staining following the standard protocol at Jiangsu Provincial Hospital of Integrated Traditional and Western Medicine (Nanjing, China).

### Determination of Hepatic Anti-oxidant Levels

The levels of total superoxide dismutase (SOD), reduced glutathione (GSH), malondialdehyde (MDA), and hepatic total-anti-oxidant capacity (T-AOC) contents were detected with their corresponding assay kits provided by Nanjing Jiancheng Bioengineering Institute (Nanjing, China), according to manufacturer instructions.

### RNA Isolation and Quantitative Real-Time Polymerase Chain Reaction

Total RNA was extracted from rat’s liver tissues by TRIzol reagent (Invitrogen Life Technologies, Carlsbad, CA, USA) following manufacturer instructions provided, 1 μg RNA was quantified using 2000 Nanodrop Spectrophotometer (Thermo Fisher Scientific, Wilmington, DE, USA) then reverse-transcribed into cDNA with PrimeScript^TM^ RT Master Mix (Takara Biotechnology, Dalian, China). Quantitative polymerase chain reaction (PCR) was executed in 20 μL volume containing 10 μL SYBR Green Supermix (Vazyme Biotech, Nanjing, China), 1 μL of cDNA, 7 μL of RNase/DNase-free water and 500 nM each primer. The thermal cycler conditions were as follows: 30 s at 95°C, followed by 40 cycles of 5 s at 95°C and 10 s at 60°C. A melting curve analysis was carried out for each reaction from 65 to 95°C. The threshold cycle at which the fluorescent signal reached an arbitrarily set threshold near the middle of the log-linear phase of amplification for each reaction was calculated, and relative quantities of each mRNA were determined. Primers used for PCR were listed in Supplementary Table S1. Gene expression was evaluated by ΔΔCT method in which glyceraldehyde-3-phosphate dehydrogenase (GAPDH) served as a reference gene.

### Western Blot Analysis

Protein samples were extracted from liver tissues with radioimmunoprecipitation assay (RIPA) buffer with phosphatase and protease inhibitors (Beyotime, China). The extracted proteins (70 μg) were quantified by bicinchoninic acid protein assay kit (Beyotime, China) and prepared with 4× sample buffer (BD Biosciences, USA). Following their separation in sodium dodecyl sulfate polyacrylamide gel electrophoresis, proteins were transferred to the polyvinylidene difluoride membranes, blocked with 5% bovine serum albumin (BSA) for 1 h and further incubated with specific primary antibodies overnight. The proteins in bands were then incubated with secondary antibodies and detected on a Bio-Rad Gel Doc XR^+^ Imaging System (CA, USA). Primary and secondary antibodies used for western blotting were listed in Supplementary Tables S2,S3, respectively.

### Terminal Deoxynucleotidyl TUNEL Staining and Immunohistochemistry

Paraffin-embedded liver sections were stained by transferase dUTP nick-end labeling (TUNEL) assay in order to identify apoptotic hepatocytes using TUNEL detection kit (KeyGEN BioTECH, Nanjing, China) following the manufacturer’s guidelines. TUNEL-stained liver samples were captured with light microscope (Olympus IX81). Immunohistochemistry testing was performed for CYP2E1 and peroxisome proliferator-activated receptors alpha (PPARα) using corresponding antibodies on hepatic tissues fixed in formaldehyde and embedded in 3 μ paraffin slices.

### Statistical Analysis

The data in all experiments are expressed as mean ± SD. Statistical comparisons were performed by one-way analysis of variance; difference between two groups was analyzed by Student’s two-tailed *t*-test using GraphPad Prism 6.0 (Graph-Pad, San Diego, CA, USA). The level of significance was set at *P*-value <0.05.

## Results

### LPS Aggravated INH Hepatotoxicity While DEX Alleviated LPS-Induced Toxicity Augmentation

**FIGURE 1:**
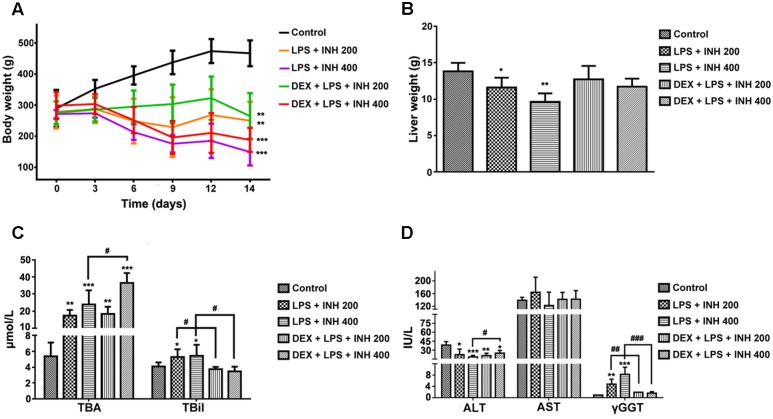
**Effects of DEX administration on liver injury parameters induced by INH/LPS co-administration.** Rats were treated with INH 200 or 400 mg/kg for 14 consecutive days, at day 14 they were given 4 mg/kg DEX 1 h earlier before they received 2 mg/kg LPS dose followed by INH 2 h later. **(A)** Effects on body weight. **(B)** Liver weight variation. **(C)** Impact on serum TBA and TBil levels. **(D)** Alterations in serum ALT, AST, and GGT levels. Data were represented as mean ± SD, *n* = 8 for each bar. ^∗^*P* < 0.05, ^∗∗^*P* < 0.01, ^∗∗∗^*P* < 0.001 versus control, ^#^*P* < 0.05, ^##^*P* < 0.01, ^###^*P* < 0.001 versus INH/LPS combination.

**FIGURE 2:**
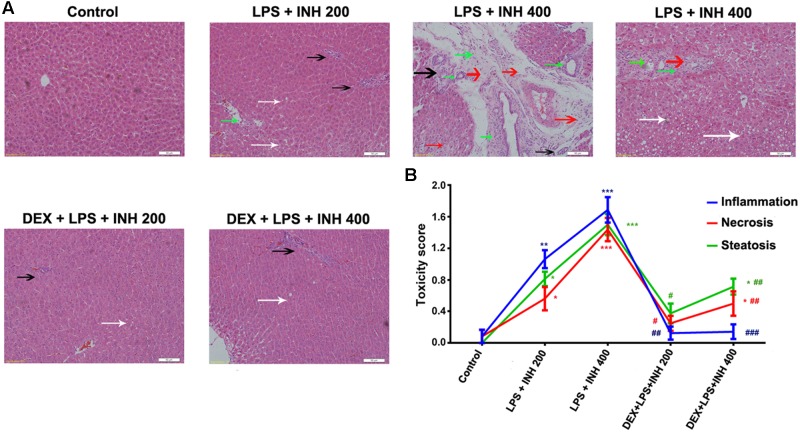
**DEX protects against INH/LPS-induced hepatic damage as indicated by liver histopathological examination.** **(A)** Liver slices were collected and subjected to staining with hematoxylin and eosin. Control group showed normal hepatocyte architecture, meanwhile INH/LPS co-treated animals liver showed sever toxicity symptoms. Inflammatory cells and inflammatory infiltration (green arrow), micro- and macrovesicular steatosis (white arrow), massive necrosis (black arrow), and hepatocellular structure loss (red arrow). On the other hand, DEX pre-treatment minimized these liver injury indicators **(B)** INH/LPS hepatotoxicity score in absence or presence of DEX. Data were represented as mean ± SD, *n* = 8 for each bar. ^∗^*P* < 0.05, ^∗∗^*P* < 0.01, ^∗∗∗^*P* < 0.001 versus control, ^#^*P* < 0.05, ^##^*P* < 0.01, ^###^*P* < 0.001 versus INH/LPS combination.

INH/LPS co-treated animals showed reduction in both total body weight and liver weight, while DEX-pretreated rats revealed insignificant partial increment in total body weight with improvement in liver weight nearly up to the control group ones (**Figures 1A,B**). Meanwhile, serum markers of liver injury including TBA, TBil, and GGT showed significant elevation in the animal groups treated by INH and LPS combination, with highest increase within INH dose 400 mg/kg. Pre-addition of DEX reduced serum TBil and GGT levels to those in the control group (i.e., significantly indifferent from control group measurement) while TBA level remained elevated, especially in the rat group that received INH 400 mg/kg (**Figures 1C,D**). In line with our previous results, serum ALT level showed significant reduction in INH/LPS-treated rats, while those receiving DEX revealed marginal increment but still beneath the control levels. Furthermore, AST levels insignificantly fluctuated in the INH/LPS co-treated rats, while administration of DEX resulted in normal control levels (**Figure 1D**). Histopathological evaluation revealed massive hepatocellular damage, necrosis, micro-and macro-vesicular steatosis accompanied with severe inflammation and inflammatory infiltration in INH/LPS-treated rats (**Figure 2A**). However, in DEX-administered rats there were no signs of inflammation, inflammatory infiltration, or steatosis rather than minor hepatocellular necrosis as observed and indicated in the histological toxicity score (**Figure 2B**).

### Hepatic Fat is Reduced in INH/LPS-Treated Rats Following DEX Administration

**FIGURE 3:**
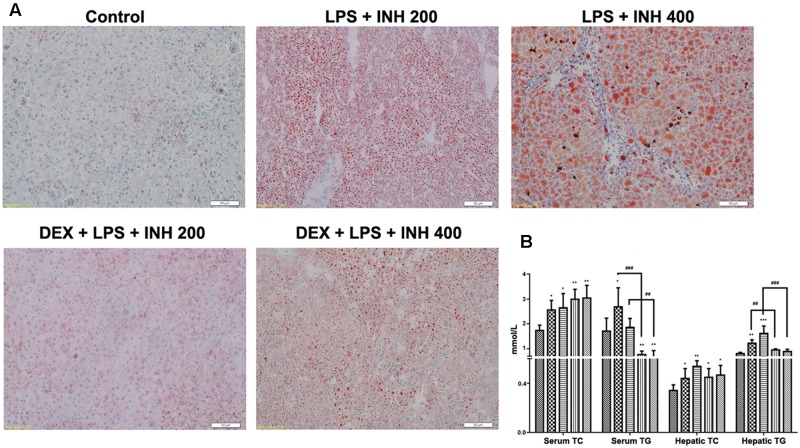
**Lipid profile analysis.** **(A)** Lipid staining in rat livers. Frozen liver sections were stained with red oil O staining; massive steatosis seen in rats received INH/LPS co-treatment whereas DEX addition decreased hepatic lipid accumulation. **(B)** Variation in both serum and hepatic lipid profile. Data were represented as mean ± SD, *n* = 8 for each bar. ^∗^*P* < 0.05, ^∗∗^*P* < 0.01, ^∗∗∗^*P* < 0.001 versus control, ^##^*P* < 0.01, ^###^*P* < 0.001 versus INH/LPS combination.

Since micro- and macro-vesicular lipid droplets had been obviously accumulated and easily detected under microscope after H&E staining, we carried out oil red O staining. As shown in **Figure 3A**, both 200 and 400 mg/kg INH plus LPS groups displayed intense dose-dependent micro- and macro-vesicular steatosis as confirmed by the strong staining, while liver slides from DEX pre-treated rats showed minor staining of lipid droplets, indicating that DEX minimizes the steatotic-induction abilities of INH/LPS co-treatment.

To validate the changes in hepatic lipid profile, both hepatic TG and TC were quantified using their corresponding quantification kits. Hepatic TC level was significantly increased in INH/LPS-treated animals, with high elevation observed in rats receiving INH 400 mg/kg (**Figure 3B**). Meanwhile, hepatic TG level also showed significant elevation in rats administered INH/LPS combination, but in rats pre-treated with DEX, their liver TG levels rendered almost to the normal control levels. Moreover, serum TC and TG levels were significantly elevated after administration of INH/LPS combination, while serum TG level significantly reduced in rats given DEX whereas serum TC levels were kept raising (**Figure 3B**).

### Hepatic Oxidative Stress-Related Parameters

**FIGURE 4:**
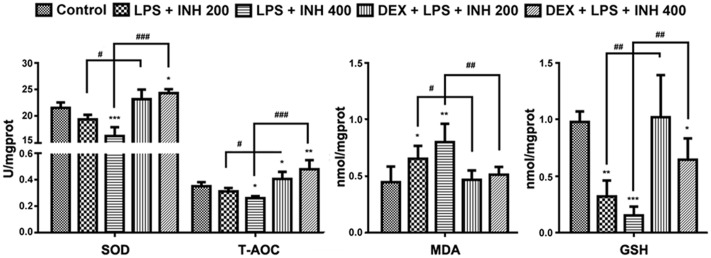
**INH/LPS caused modifications in oxidative stress and hepatic anti-oxidant defense mechanisms while DEX pre-administration helped in elevation of liver anti-oxidant defense.** Changes in SOD, T-AOC, MDA, and GSH levels were measured by their relevant kits. Data were represented as mean ± SD, *n* = 8 for each bar. ^∗^*P* < 0.05, ^∗∗^*P* < 0.01, ^∗∗∗^*P* < 0.001 versus control, ^#^*P* < 0.05, ^##^*P* < 0.01, ^###^*P* < 0.001 versus INH/LPS combination.

Oxidative stress created from the imbalance between free radicals’ generation and removal following INH treatment has being proposed to be one of the causative mechanisms by which INH could produce hepatic damage (Shen et al., 2007; Rao et al., 2012). As indicated in **Figure 4**, INH/LPS combination showed a significant reduction in both SOD and T-AOC levels with maximum reduction observed at animal group receiving INH 400 mg/kg, which were significantly improved after addition of DEX. Furthermore, MDA is one of the oxidative stress factors being produced as a result from lipid peroxidation, MDA determination showed significant elevation in INH/LPS-treated rats with highest elevation observed in INH dose 400 mg/kg, while DEX pre-administered groups, their MDA levels returned to the normal control ones.

Liver GSH levels exhibited significant inhibition especially in INH 400 mg/kg plus LPS. Meanwhile, rats pre-treated with DEX revealed improvement in GSH levels. These results indicated that, hepatic anti-oxidant abilities have been raised due to the DEX pretreatment which led to minimum hepatocellular damage and reduced the liver injury.

### Hepatic Gene Expressions in INH/LPS-Induced Liver Injury

**FIGURE 5:**
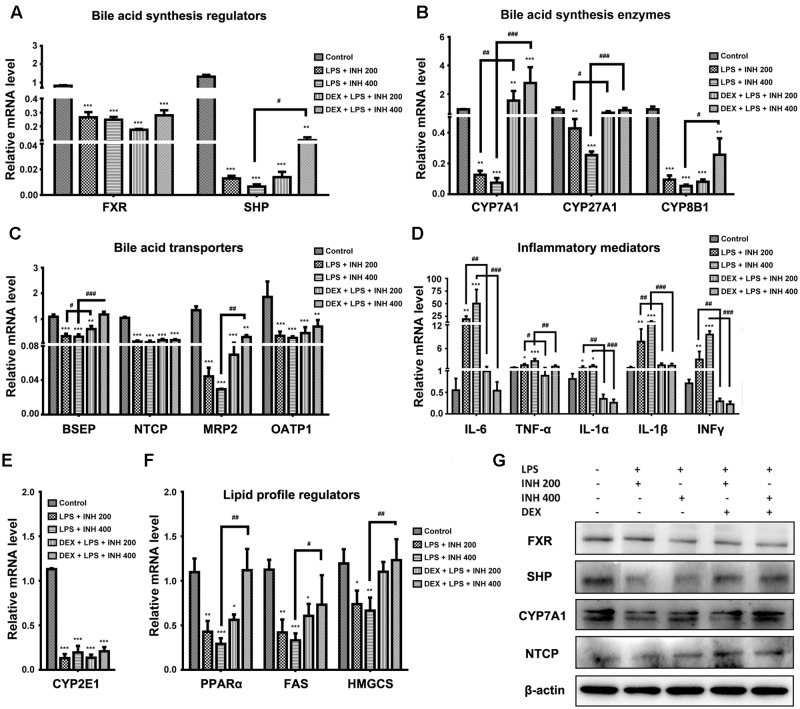
**Effects of INH/LPS combination on different targeted genes and proteins profile in absence or presence of DEX.** **(A)** Bile acid regulators FXR and SHP expression. **(B)** Expression of bile acid synthesis enzymes CYP7A1, CYP27A1, and CYP8B1. **(C)** Bile acid transporters BSEP, NTCP, MRP_2_, and OATP1 expression. **(D)** Inflammatory mediators IL-6, TNFα, IL-1α, IL-1β, and INFγ expression. **(E)** Expression of CYP2E1. **(F)** Lipid profile regulators PPARα, FAS, and HMGCS expression. Data were represented as mean ± SD, *n* = 8 for each bar. ^∗^*P* < 0.05, ^∗∗^*P* < 0.01, ^∗∗∗^*P* < 0.001 versus control, ^#^*P* < 0.05, ^##^*P* < 0.01, ^###^*P* < 0.001 versus INH/LPS combination, GAPDH was set as reference control gene. **(G)** Immunoblotting analysis of different targeted proteins following INH/LPS with or without DEX treatment, β-actin considered as loading control.

Due to the elevation of serum bile acids parameters mainly TBA, TBil, and GGT in rats receiving INH/LPS combination, we carried out further investigation of the relevant genes associated with bile acids. As indicated in **Figure 5A**, both farnesoid X receptor (FXR) and small heterodimer partner (SHP) expressions were significantly repressed in INH/LPS-treated groups, DEX pretreatment did not restore their corresponding control levels but it clearly affected expressions of CYP7A1, CYP27A1, and CYP8B1, the genes responsible for bile acid synthesis, which were significantly over-expressed in animal groups receiving DEX (**Figure 5B**). These genes significant over-expressions were positively correlated with the observed serum TBA elevation.

In the meantime, the repression of bile acid transporters bile salt export pump (BSEP), Na^+^-taurocholate co-transporting polypeptide (NTCP), organic anion-transporting polypeptide 1 (OATP1) and multi-drug resistance protein (MRP_2_) seen following INH/LPS treatment also indicated the accumulated levels of toxic bile acids in the hepatocytes. Surprisingly, DEX pretreatment significantly elevated BSEP and MRP_2_ expression indicated the reduction of the hepatocyte toxic bile acids accumulation, but DEX showed no improving signs on NTCP and OATP1 expressions (**Figure 5C**). Meanwhile, DEX administration also caused MRP_3_ over-expression (data not shown) which could further explained the elevated serum bile acids level noticed in rats received both DEX and INH/LPS combination.

Although immune system’s role have been neglected in the pathogenesis of INH-induced liver toxicity due to the absence of fever, rash, eosinophilia, and rapid start of INH toxicity upon dose resuming (Metushi et al., 2016), but more recent findings support the relationship between INH toxicity and immune system (Salazar-Paramo et al., 1992; Metushi et al., 2011; Metushi et al., 2014). Consistently with these recent findings, our results demonstrated that, the inflammatory mediator cytokines as interleukin 6 (IL-6), tumor necrosis factor alpha (TNF-α), IL-1α, IL-1β, and interferon gamma (INFγ) were significantly over-expressed in INH/LPS-treated rats whereas DEX pre-treatment significantly reversed their levels to their corresponding control group values (**Figure 5D**) therefore, eliminating the augmented role of the LPS through prevention of the inflammatory stress.

Many researchers, including our previous report, focused on the core role played by CYP2E1 in INH toxicity (Yue et al., 2004; Cheng et al., 2013; Shayakhmetova et al., 2015; Hassan et al., 2016), we found a positive correlation between INH hepatotoxicity and CYP2E1. Spectacularly, CYP2E1 at its gene expression level almost dwindled in all INH/LPS-treated rats, even those already receiving DEX (**Figure 5E**). Meanwhile, hepatic lipid accumulation as evidenced by analysis of hepatic TC and TG and red oil O staining led us to study the change in the PPARα, which is highly expressed in liver and accumulating evidences report the vital role of PPARα in the regulation of hepatic lipid profile (Silva et al., 2015). **Figure 5F** also displayed the significant repression of PPARα in INH/LPS groups while in rats pre-treated with DEX; their PPARα expression was significantly elevated. Furthermore, the enzymes responsible for synthesis of lipids, mainly fatty acid synthase (FAS) and hydroxymethylglutaryl-CoA synthase (HMGCS) revealed significant reduction in INH/LPS groups, which might be attributed to the severe accumulation of hepatic lipids, while those receiving DEX their FAS and HMGCS expression levels were nearly normal (**Figure 5F**).

### Corresponding Protein Expressions Analyzed by Both Western Blot and Immunohistochemistry

Associated with their relevant expressed genes, protein levels of FXR, SHP, and NTCP were reduced in INH/LPS-treated rats whereas their expression levels were elevated in rats received DEX, except for FXR (**Figure 5G**). Meanwhile, CYP7A1 protein expression was reduced in the absence of DEX, while in the presence of DEX, INH/LPS-treated animals showed supreme protein level, which was clearly in match with its gene expression level and further explained the elevated serum TBA observed in these rats.

**FIGURE 6:**
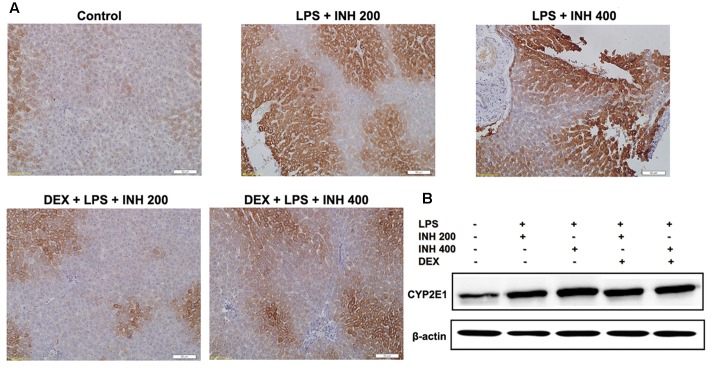
**Variations in hepatic CYP2E1 protein expression.** **(A)** INH/LPS treatment elevated CYP2E1 expression, especially in rats received INH 400 mg/kg (deep brown color). Moreover, DEX administration marginally reduced CYP2E1 expression probably through elimination of LPS-enhancing effects, as indicated by the decreased color intensity. **(B)** CYP2E1 immunoblotting analysis, β-actin used as internal control.

CYP2E1 protein level showed pronounced elevation in animals receiving INH/LPS combination with highest increment observed in rats received LPS/INH 400 mg/kg. Unexpectedly DEX pre-treatment did not completely block CYP2E1 protein expression, but only caused marginal reduction in CYP2E1 protein expression, fortifying the fact that, LPS only over-activated and sensitize the liver toward CYP2E1 effects, therefore blocking of LPS will not lead to CYP2E1 inhibition. Interestingly, CYP2E1 immunohistochemistry results revealed that, in line with the protein levels, INH/LPS-treated rats had more intense coloration reflected the hepatocellular CYP2E1 accumulation. In contrast, rats received DEX pre-treatment, their hepatocyte CYP2E1 level was significantly reduced as indicated by more fading colors (**Figures 6A,B**).

**FIGURE 7:**
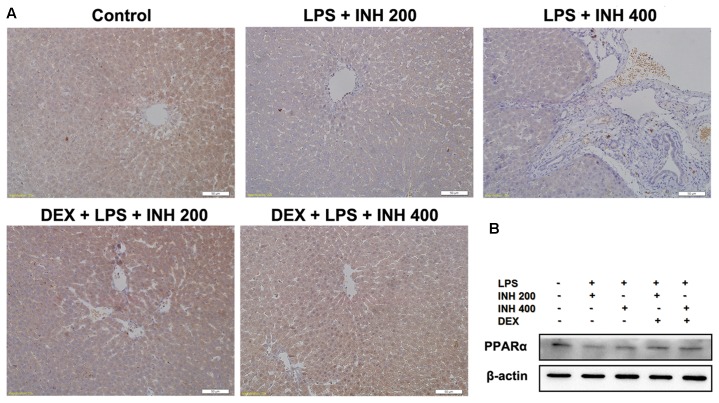
**Alterations of PPARα expression in rat liver.** **(A)** Photomicrograph of PPARα in rat liver immunohistochemistry, control group showed brown staining (color indicating PPARα) while INH/LPS-treated rats their PPARα expression was significantly reduce as indicated by color fading. DEX pre-treatment ameliorate PPARα expression after INH/LPS treatment. **(B)** Total liver protein was probed for PPARα, β-actin considered as loading control.

We also focused on the protein level of PPARα in liver after INH/LPS treatment in order to verify the PCR result. Western blot and immunohistochemistry experiments showed that, PPARα protein expression was significantly down-regulated, but its expression was up-regulated in the DEX pre-administered groups (**Figures 7A,B**).

### DEX Prevented Hepatocellular Apoptosis in INH/LPS Model

**FIGURE 8:**
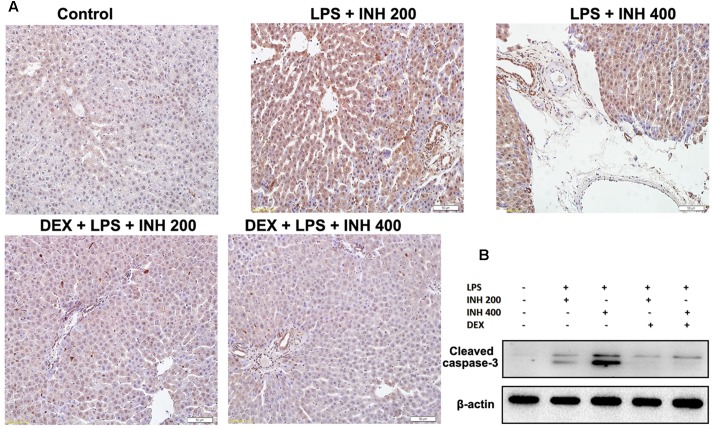
**INH/LPS treatment caused apoptosis in hepatic tissues, meanwhile DEX prevented hepatocellular apoptosis in INH/LPS.** **(A)** TUNEL assay was conducted on liver slices; INH/LPS co-administration caused obvious positive TUNEL results (intensity of brown color indicating apoptotic action). In the meantime, DEX restrained INH/LPS tendency to induce apoptosis (absence of brown color). **(B)** Representative western blot analysis of cleaved caspase-3 (β-actin used as internal control).

As experimentally evident, most of INH liver damage came through apoptotic induction in hepatocytes and breaks of DNA strands (Lee et al., 2013; Perwitasari et al., 2015). As displayed in **Figure 8A** after TUNEL assay application, no observation of hepatocyte apoptosis in the control group, whereas following INH/LPS co-treatment, rat hepatocytes were stained TUNEL positive, however, addition of DEX led to a decrease in this apoptotic activity, revealing that DEX suppressed the apoptotic-stimulating effect of LPS on INH toxicity. This finding was further fortified by analysis of the indicator of cellular apoptosis, hepatic cleavage caspase-3 protein levels, in which groups received INH/LPS co-therapy manifested higher protein expression which was not present after DEX addition (**Figure 8B**).

## Discussion

Despite its ability to generate liver toxicity, INH remains an indispensable first-line drug for treatment of tuberculosis especially latent tuberculosis, but the exact mechanisms by which INH induces liver injury are not fully understood (Boelsterli and Lee, 2014; Metushi et al., 2016). Our group early studies succeed to establish a rat model of INH hepatotoxicity employing the inflammatory stress theory in which non-injurious doses of an inflammatory-mediating agent (LPS) augmented the toxicity of INH (Su et al., 2014; Hassan et al., 2016). In order to confirm our previous findings, we conducted the same preceding 14 days experiment with addition of DEX, a potent glucocorticoid anti-inflammatory agent, to alleviate LPS potentiation activity and to relieve the associated symptoms of INH hepatic injury, which have been extensively reported to be indistinguishable from hepatitis symptoms (Bjornsson et al., 2007).

We again successfully established INH/LPS-induced liver injury model as verified by body weight reduction, blood biochemical parameters and histopathology, DEX pretreatment assisted in restoration of INH/LPS-treated rat’s livers to their corresponding normal control as evidenced by reduced serum levels of TBil, GGT as well as histological examination. Due to the fact that serum ALT and AST levels, which are considered as the cornerstone indicators of liver damage, were unreliable in judging INH-induced liver injury because of their time-dependent variations (Karthikeyan, 2004; Metushi et al., 2012; Jahan et al., 2015), serum TBA, TBil, and GGT levels might be perfect substitutes as marker diagnostic parameters for INH hepatotoxicity. Based on this assumption, DEX helped in prevention of INH-induced liver damage as indicated by TBil and GGT reduction. However, our results revealed that DEX-pretreated rats, especially those received INH 400 mg/kg, their serum TBA levels were significantly elevated. This elevation was coherent with the up-regulation of the bile acid synthesizing genes CYP8B1, CYP7A1, and CYP27A1. Interestingly, these bile acid synthesizing genes showed the same repressive effects as previously observed in the animals administered INH/LPS combination (Hassan et al., 2016), mimicking their feedback inhibition theory due to accumulation of extreme hepatocellular bile acids levels (Kunne et al., 2014).

As being considered the cornerstone rate-limiting enzyme in the synthesis of bile acids from catabolism of cholesterol, CYP7A1 expression regulates the bile acids level (Fan et al., 2015) while CYP27A1 acts as the alternative pathway in the bile acids production (Zhou and Hylemon, 2014). Furthermore, DEX-induced BSEP over-expression could also participates as a secondary cause for serum bile acid elevations seen in DEX-treated animals. Glucocorticoids, including DEX, are known to increase bile acid production (Lu et al., 2012) through augmentation of synthesizing enzymes CYP7A1 and CYP27A1 (Princen et al., 1989; Liu et al., 2008) and elevate expressions for both BSEP and MRP_3_ (Mörk et al., 2016) that enhance bile acid secretion which end up in serum accumulation of bile acids. Moreover, endotoxin mainly down-regulates MRP_2_ expression both at mRNA and protein levels (Kubitz et al., 1999) therefore, DEX-induced MRP_2_ up-regulation could be attributed to its anti-endotoxin activity which counteracts the LPS-induced MRP_2_ repression. Although glucocorticoids treatment is usually associated with NTCP up-regulation (Xiao et al., 2016), DEX pre-administration only succeed in elevation of the NTCP protein expression while no effect was observed on mRNA gene expression level.

Accretion of toxic bile acid levels in the hepatocytes could induce oxidative stress that promotes liver damage (Nomoto et al., 2009). Moreover, inflammation, either induced by toxic bile acids (Allen et al., 2011), INH (Chen et al., 2011, 2013) or LPS administration (Sekine et al., 2010) and hepatic CYP2E1 (Warren et al., 1999), further potentiates the hepatocyte oxidative stress which was very obvious in our results through elevation of MDA level and the reduction of GSH, SOD, and T-AOC levels. However, pre-addition of DEX assisted in improving the hepatic anti-oxidant mechanisms mainly via inhibition of LPS-induced intracellular ROS production (Huo et al., 2011). On the other hand, INH/LPS combination obviously provoked liver damage, necrosis, and hepatocellular apoptosis as indicated by histopathological evaluation, elevated cleavage caspase-3 level and TUNEL assay whereas combining DEX with INH/LPS help protects from liver injury. One assumption of this protection is DEX strengthening of hepatic anti-oxidant mechanisms that prevent hepatocyte oxidative stress-induced damage, also its ability to protect hepatocytes from toxic bile acids accumulation. Moreover, DEX is believed to prevent both necrosis and apoptosis, not only in liver but also in other tissues such as the kidney, through inhibition of caspase-3 activity and up-regulation of anti-apoptotic protein Bcl-xL (Moller et al., 2015). Therefore, inhibition of caspase-3 activity and induction of Bcl-xL not only prevent tissue necrotic and apoptotic potentials, but also exerts a potent anti-inflammatory response that help in suppressing immune-system mediated liver damage (Malhi et al., 2006).

Furthermore, DEX down-regulates the inflammatory mediators such as IL-6, TNF-α, IL-1α, IL-1β, and INFγ which were over-expressed in INH/LPS-treated rats and potentiate the INH/LPS apoptotic-inducing capabilities. This potent anti-inflammatory effect could be responsible for DEX-mediated liver protection as many recent studies are in agreement with this concept (Hsieh et al., 2004, 2006; Yubero et al., 2009). This DEX protection is not only limited to the liver tissues, but also brain tissues (Pang et al., 2012; Du et al., 2014) and inflammatory-induced microcirculation dysfunction (Cui et al., 2015). Surprisingly, Yubero et al. (2012) concluded that DEX although achieved a potent inflammatory cytokines inhibition, but it failed to protect the lung tissue injury.

Alongside it is central key role in ROS generation, CYP2E1 play an essential role in INH/LPS-induced hepatotoxicity. INH and its metabolites are responsible for hepatic CYP2E1 induction (Yue et al., 2004; Cheng et al., 2013). Meanwhile, LPS and CYP2E1 shared a unique relationship in which bacterial LPS not only stimulating CYP2E1 expression, but also increases the hepatic sensitivity toward CYP2E1 that further exaggerates INH/LPS toxicity (Lu and Cederbaum, 2010; Cederbaum et al., 2012; Shayakhmetova et al., 2015). Our results clearly indicated that DEX assisted in marginal reduction of CYP2E1 expression, but its capability to fully block CYP2E1 was unsuccessful. This marginal reduction could be ascribed to DEX-mediating LPS inhibition. Moreover, CYP2E1 has the tendency to heighten the bile acid level through an efficacious role in bile acid synthesis pathway (Cheng et al., 2013), which further explained the elevated serum TBA level spotted in DEX pre-treated animals. Previous studies extensively reported that CYP2E1 expression is totally not affected by DEX (Monostory and Vereczkey, 1994; Burkina et al., 2013), however, in contradiction to these studies, others documented the DEX-induced CYP2E1 expression potential (Sampol et al., 1997; Zerilli et al., 1998; Tamasi et al., 2001). Regardless these controversial findings about DEX effect on CYP2E1 expression, our experimental outcomes distinctly abolish the hypothesis that DEX could entirely block CYP2E1 expression. Our experiments indicate that partial liver injury seen in rat groups received INH/LPS combined with DEX might be due to the CYP2E1-inhibition inabilities of DEX.

**FIGURE 9:**
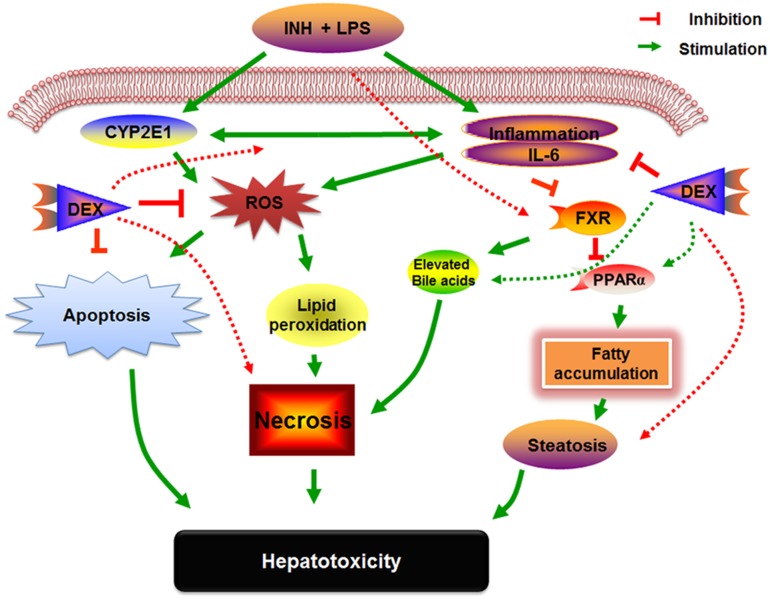
**Schematic presentation highlighting the proposed mechanisms by which INH/LPS-induced hepatotoxicity and the DEX mechanistic intervention for liver protection in INH/LPS liver injury model**.

Researchers are very familiar with the potential activities of PPARα in liver and other extremely metabolic-active tissues, those activities extend between adipocyte differentiation, fatty acid oxidation, inflammation as well as glucose and lipid metabolism (Corton et al., 2014). On the other hand, PPARα and its agonist positively adjust the expression of the key regulator genes involved in hepatic lipogenesis such as FAS and HMGCS (Kunne et al., 2014; Pawlak et al., 2015). Furthermore, PPARα has a participatory part in hepatic preservation from inflammation (Stienstra et al., 2007), oxidative stress through CYP2E1 up-regulation (Okiyama et al., 2009), hepatic fat accumulation and hepatocellular apoptosis (Abdelmegeed et al., 2011). Our established INH/LPS hepatotoxicity rat model revealed repression of PPARα at both mRNA and protein levels which were accompanied by elevated serum and hepatic TC and TG levels as well as micro- and macro-vesicular lipid vacuoles as shown in the liver histopathology and oil red O staining. In contrast, DEX pre-treated rats showed elevated levels of PPARα, that reflected reduction of hepatic inflammation, increased hepatic anti-oxidant activity and liver steatosis. Our comprehensive examinations of DEX-administration to INH/LPS-treated rats can be entirely interpreted in **Figure 9**.

In conclusion, this study elucidated the DEX-preventive capabilities on INH/LPS-induced hepatotoxicity model. These data strengthened the fact that bacterial inflammation as well as CYP2E1 over-expression initiate and propagate INH-induced liver injury while potent anti-inflammatory agents such as DEX could strongly prevent the inflammatory stress. These findings potentiate the clinical applications of DEX co-administration with INH therapy in order to reduce the potential incidences of hepatotoxicity. Although DEX significantly alleviates both inflammatory and oxidative stress, but it showed no major effect on the hepatic CYP2E1 expression. Therefore, additional studies using specific CYP2E1 inhibitors or other techniques are needed to further explain CYP2E1 participation in INH-induced liver damage.

## Author Contributions

HH, HG, ZJ, and LZ participated in research design. HH, HG, BY, and DP-P carried out the experiments. HH performed data analysis. HH, BY, and ZJ wrote the manuscript.

## Conflict of Interest Statement

The authors declare that the research was conducted in the absence of any commercial or financial relationships that could be construed as a potential conflict of interest.
