# Supplementary material for: Dexamethasone Pretreatment Alleviates Isoniazid/Lipopolysaccharide Hepatotoxicity: Inhibition of Inflammatory and Oxidative Stress
Source: Front Pharmacol. 2017 Mar 15;8:133. doi: 10.3389/fphar.2017.00133 (PMC5350150; doi:10.3389/fphar.2017.00133)
Supplement: Supplementary file 1 [file Table_1.DOCX]

**Table 1: Primers for Real-time PCR**

| Gene | Primer orientation | | Nucleotide sequence | NCBI Gene ID number | | |  |
| --- | --- | --- | --- | --- | --- | --- | --- |
| *GAPDH* | | forward | 5’- ATGGAGAAGGCTGGGGCTCACCT-3’ | | 24383 |  |  |
|  |  | reverse | 5’-AGCCCTTCCACGATGCCAAAGTTGT-3’ | |  |  |  |
| *FXR* | | forward | 5’- TGGACTCATACAGCAAACAGAGA-3’ | 60351 | | | |
|  | | reverse | 5’- GTCTGAAACCCTGGAAGTCTTTT-3’ |  | | | |
| *SHP* | | forward | 5’- ACCTGCAACAGGAGGCTCACT-3’ | 117274 | | | |
|  | | reverse | 5’- TGGAAGCCATGAGGAGGATTC-3’ |  | | | |
| *CYP7A1* | | forward | 5’- CAGGGAGATGCTCTGTGTTCA-3’ | 25428 | | | |
|  | | reverse | 5’- AGGCATACATCCCTTCCGTGA-3’ |  | | | |
| *CYP27A1* | | forward | 5’- GGAAGGTGCCCCAGAACAA-3’ | 301517 | | | |
|  | | reverse | 5’- GCGCAGGGTCTCCTTAATCA-3’ |  | | | |
| *CYP8B1* | | forward | 5’- GTACACATGGACCCCGACATC-3’ | 81924 | | | |
|  | | reverse | 5’- GGGTGCCATCAGGGTTGAG-3’ |  | | | |
| *BSEP* | | forward | 5’- CAACGCATTGCTATTGCTCG-3’ | 83569 | | | |
|  | | reverse | 5’- CTTCTGGATGGTGGACAAACG-3’ |  | | | |
| *NTCP* | | forward | 5’- GCATGATGCCACTCCTCTTATAC-3’ | 24777 | | | |
|  |  | reverse | 5’- TACATAGTGTGGCCTTTTGGACT-3’ |  |  |  |  |
| *MRP_2_* | | forward | 5’- CAGTCACGGCTTCCTTTCTG-3’ | 25303 | | | |
|  | | reverse | 5’- AGGTTTCCGCTGGGACTTCT-3’ |  | | | |
| *OATP1* | | forward | 5’- GGCTTTTTGGTCTGTGCAGG-3’ | 50572 | | | |
|  | | reverse | 5’- CACCTTGTGTTGCAGTCAGC-3’ |  | | | |
| *TNFα* | | forward | 5’- TGGTGGGAGACATTGGAGAT-3’ | 24835 | | | |
|  | | reverse | 5’- GCGGCTTGATAAACACATCA-3’ |  | | | |
| *IL-6* | | forward | 5’- AGAGACTTCCAGCCAGTTGC-3’ | 24498 | | | |
|  | | reverse | 5’- AGTCTCCTCTCCGGACTTGT-3’ |  | | | |
| *IL-1α* | | forward | 5’- CCTCGTCCTAAGTCACTCGC-3’ | 24493 | | | |
|  | | reverse | 5’- GGCTGGTTCCACTAGGCTTT-3’ |  | | | |
| *IL-1β* | | forward | 5’- CCTGTTCTTTGAGGCTGACA-3’ | 24494 | | | |
|  | | reverse | 5’- GCTGTGAGATTTGAAGCTGGA-3’ |  | | | |
| *INFγ* | | forward | 5’- ACAACCCACAGATCCAGCAC-3’ | 25712 | | | |
|  | | reverse | 5’- CCAGAATCAGCACCGACTCC-3’ |  | | | |
| *CYP2E1* | | forward | 5’- GTGGTCCTGCATGGCTACA-3’ | 25086 | | | |
|  | | reverse | 5’- ACCTCCGCACATCCTTCC-3’ |  | | | |
| *PPARα* | | forward | 5’- GTCCTCTGGTTGTCCCCTTG-3’ | 25747 | | | |
|  | | reverse | 5’- GTCAGTTCACAGGGAAGGCA-3’ |  | | | |
| *FAS* | | forward | 5’- GACTACAGACAACAGCAACC-3’ | 50671 | | | |
|  | | reverse | 5’- CTCAGACAGGCACTCAGG-3’ |  | | | |
| *HMGCS* | | forward | 5’- GGACCAACCTTCTACCTCAG-3’ | 29637 | | | |
|  | | reverse | 5’- ACAACTCACCAGCCATCAC-3’ |  | | | |
