# Supplementary material for: Dexamethasone Pretreatment Alleviates Isoniazid/Lipopolysaccharide Hepatotoxicity: Inhibition of Inflammatory and Oxidative Stress
Source: Front Pharmacol. 2017 Mar 15;8:133. doi: 10.3389/fphar.2017.00133 (PMC5350150; doi:10.3389/fphar.2017.00133)
Supplement: Supplementary file 2 [file Table_2.DOCX]

**Table 2: Primary antibodies for western blot**

| **Primary antibodies** | | | |
| --- | --- | --- | --- |
| Antibody | Catalog number | Company |  |
| *FXR* | bs-12867R | Bioss, Atlanta, GA, USA |  |
| *SHP* | sc-30169 | Santa Cruz, CA, USA |  |
| *CYP7A1* | sc-25536 | Santa Cruz, CA, USA |  |
| *NTCP* | sc-98485 | Santa Cruz, CA, USA |  |
| *CYP2E1* | ab19140 | Abcam, Cambridge, UK |  |
| *PPARα* | sc-9000 | Santa Cruz, CA, USA |  |
| *Cleaved caspase 3* | 9661 | Cell Signaling Technology (CST) Danvers, MA, USA |  |
| *β-actin* | sc-69879 | Santa Cruz, CA, USA |  |
