# Supplementary material for: Dexamethasone Pretreatment Alleviates Isoniazid/Lipopolysaccharide Hepatotoxicity: Inhibition of Inflammatory and Oxidative Stress
Source: Front Pharmacol. 2017 Mar 15;8:133. doi: 10.3389/fphar.2017.00133 (PMC5350150; doi:10.3389/fphar.2017.00133)
Supplement: Supplementary file 3 [file Table_3.DOCX]

**Table 3: Secondary antibodies for western blot**

| **Secondary antibodies** | | | |
| --- | --- | --- | --- |
| Antibody | Catalog number | Company |  |
| Goat anti-mouse | ab6789 | Abcam, Cambridge, UK |  |
| Goat anti-rabbit | ab6721 | Abcam, Cambridge, UK |  |
| Rabbit anti-goat | ab6741 | Abcam, Cambridge, UK |  |
